# Quo Vadis HTA for Medical Devices in Central and Eastern Europe? Recommendations to Address Methodological Challenges

**DOI:** 10.3389/fpubh.2020.612410

**Published:** 2021-01-08

**Authors:** Rita Daubner-Bendes, Sándor Kovács, Maciej Niewada, Mirjana Huic, Michael Drummond, Oriana Ciani, Carl Rudolf Blankart, Olena Mandrik, Aleksandra Torbica, John Yfantopoulos, Guenka Petrova, Malwina Holownia-Voloskova, Rod S. Taylor, Maiwenn Al, Oresta Piniazhko, László Lorenzovici, Rosanna Tarricone, Antal Zemplényi, Zoltán Kaló

**Affiliations:** ^1^Syreon Research Institute, Budapest, Hungary; ^2^Centre for Health Technology Assessment, University of Pécs, Pécs, Hungary; ^3^Department of Experimental and Clinical Pharmacology, Medical University of Warsaw, Warsaw, Poland; ^4^HTA/EBM Consulting Centre, Zagreb, Croatia; ^5^Centre for Health Economics, University of York, York, United Kingdom; ^6^Centre for Research on Health and Social Care Management, SDA Bocconi School of Management, Milan, Italy; ^7^Evidence Synthesis and Modelling for Health Improvement, College of Medicine and Health, Institute of Health Research, University of Exeter, Exeter, United Kingdom; ^8^KPM Center for Public Management, University of Bern, Bern, Switzerland; ^9^sitem-insel AG, Swiss Institute for Translational and Entrepreneurial Medicine, Bern, Switzerland; ^10^School of Health and Related Research, Health Economics and Decision Science, The University of Sheffield, Sheffield, United Kingdom; ^11^Department of Social and Political Science, Bocconi University, Milan, Italy; ^12^School of Economics and Political Science, University of Athens, Athens, Greece; ^13^Department of Social Pharmacy and Pharmacoeconomics, Faculty of Pharmacy, Medical University of Sofia, Sofia, Bulgaria; ^14^Health Technology Assessment Department, State Budgetary Institution “Research Institute for Healthcare Organization and Medical Management of Moscow Healthcare Department”, Moscow, Russia; ^15^MRC/CSO Social and Public Health Sciences Unit and Robertson Centre for Biostatistics, Institute of Health and Well Being, University of Glasgow, Glasgow, United Kingdom; ^16^Erasmus School of Health Policy and Management, Erasmus University Rotterdam, Rotterdam, Netherlands; ^17^HTA Department of State Expert Centre of Ministry of Health of Ukraine, Kyiv, Ukraine; ^18^G. E. Palade University of Medicine, Pharmacy, Science and Technology, Tirgu Mures, Romania; ^19^Syreon Research Romania, Tirgu Mures, Romania; ^20^Division of Pharmacoeconomics, Faculty of Pharmacy, University of Pécs, Pécs, Hungary; ^21^Centre for Health Technology Assessment, Semmelweis University, Budapest, Hungary

**Keywords:** medical device (MD), health technology assessement (HTA), methodological challenge, transferability, value assessment, Central-Eastern Europe (CEE), real world evidence (RWE)

## Abstract

**Objectives:** Methodological challenges in the evaluation of medical devices (MDs) may be different for early and late technology adopter countries, as well as the potential health technology assessment (HTA) solutions to tackle them. This study aims to provide guidance to Central and Eastern European (CEE) countries on how to address key challenges of HTA for MDs with special focus on the transferability of scientific evidence.

**Methods:** As part of the COMED Horizon 2020 project, a comprehensive list of issues related to MD HTA were identified based on a targeted literature review. Health technology assessment issues which pose a greater challenge or require different solutions in late technology adopter countries were selected. Draught recommendations to address these issues were developed and discussed in a focus group. The recommendations were then validated with a wider group of experts, including HTA and reimbursement decision makers from CEE countries in May and June 2020.

**Results:** A consolidated list of 11 recommendations were developed in 3 major areas: (1) clinical value assessment, focusing on the use of joint EU work, relying on real-world evidence, use of coverage with evidence development schemes, transferring evidence from foreign countries and addressing the challenges of learning curve and centre effect; (2) economic value assessment, covering cost calculation of complex medical devices and transferability of economic evaluations of MDs; (3) HTA processes, related to the frequent product modifications and various indications of MDs.

**Conclusions:** Central and Eastern European countries with limited resources for conducting HTA, can benefit from HTA methods and evidence generated in early technology adopter countries. Considering the appropriate reuse of international HTA materials, late technology adopter countries can still implement HTA, even for MDs, which have a more limited evidence base compared with pharmaceuticals.

## Introduction

The use of HTA to improve the evidence base of health policy decisions has been increasing across Europe. In almost all countries where HTA has been introduced, it is mainly used to support public coverage decisions of new pharmaceuticals, partly because the availability of scientific evidence for medicines is quite substantial compared with other technologies. However, despite challenges (1), HTA has also been increasingly used to support coverage decision of MDs, which necessitates specific methodological guidelines for MDs (2).

Development and improvement of HTA methodologies is on the top agenda of the European Union (EU). The EU addressed the topic in several large scale projects financed through 7th Framework and the Horizon 2020 Research Programmes: the MedtechHTA project (3) was financed through 7th Framework Research Program, and the ongoing COMED project is financed through the Horizon 2020 Program. The COMED (Pushing the boundaries of Cost and Outcome analysis of Medical Technologies) project has multiple objectives. First, it aims to improve methods for economic evaluation for medical devices by addressing most relevant challenges in HTA of medical devices, second, to investigate health system performance through analysis of variation in access to medical technologies across different geographical areas; and finally, to strengthen the use of economic evaluation of MDs in policy making (4).

The COMED project put emphasis on extending the current knowledge on the transferability of HTA methodologies and reports for MDs especially across countries with different economic status. This is highly needed, as HTA implementation roadmaps of higher income Western European (WE) countries may not be applicable in lower income European countries, especially in Central and Eastern Europe for several reasons. The health status of population in Central and Eastern European (CEE) countries is significantly worse compared with WE countries (5, 6), which indicates greater demand for health technologies with substantial incremental health gain. On the other hand, the availability of public health care budgets to cover new technologies—including MDs—is more limited in CEE countries. Therefore, these countries incur a higher opportunity cost for inappropriate, not evidence-based policy decisions.

Central and Eastern European countries have more limited financial capacities for health technology assessment, furthermore, there is lack of sufficient human resources and expertise due to scarcity of post-graduate programs (7). Due to the lower market potential related to budget limitations, manufacturers tend to launch their new MDs later in CEE than in WE countries, which creates an opportunity for making use of accessible HTA reports prepared by influential HTA institutes in early adopter countries of new health technologies.

While the methodological challenges of WE and CEE countries in the evaluation of MDs may be similar, potential HTA solutions may be different due to the abovementioned reasons. This document focuses on lower income and/or small-size European countries which are late adopters of medical devices due to their limited market potential (8), with special focus on CEE EU member states. Our objectives are (1) to explore priority issues in conducting HTA for MDs, which are either specific to CEE countries, or are present in all EU countries but present greater challenges, or require different solutions in CEE countries; and (2) to provide guidance to CEE countries on how to address these challenges of MDs with special focus on the transferability of scientific evidence.

Given these objectives, this document focuses on HTA challenges that are more specific to medical devices as compared with pharmaceuticals. Also, HTA challenges that are equally relevant, and require similar solutions in early and later technology adopter countries, are not in our scope. Finally, it is not our intention to provide recommendations on how priority setting should be implemented at the national level, including which MDs should be selected for mandatory HTA prior to policy decisions and what should be the role of HTA in the pricing and reimbursement of MDs.

On the other hand, MDs usually subjected to HTA in the majority of countries are those which can deliver incremental effectiveness or safety to patients. Therefore, our recommendations may not be relevant to those decision support MDs (e.g., shared decision-making tools, digital platforms), which do not promise direct health gain.

## Materials and Methods

A targeted literature review was conducted in Scopus to identify a comprehensive list of issues of medical device HTA. A snowball method was also used to identify further relevant studies among the references of papers with full text review. The search strategy used with the date of the search and number of hits are summarised in Supplementary Material 1.

In a series of iterative brainstorming sessions, the results of the literature review were discussed with senior experts with experience in the HTA of MDs. Structured discussion was conducted about all issues where the experts had to judge which issues are relevant for European countries which are typically late adopters of MDs. The relevant issues were selected by considering certain requirements, such as non-redundancy, non-overlap and preference independence. Issues that were equally relevant to all European countries or do not require specific recommendations in CEE were excluded. The iterative discussion was continued until all participants agreed with the inclusion of the specific issue.

The iterative discussions were also used to propose draught recommendations on how to manage the identified issues. To provide sufficient details while preserving clarity, each recommendation had a short form and a detailed description.

The original plan was to refine and validate the draught list of recommendations in consecutive meetings with senior international experts. However, in the COVID-19 outbreak period we had to convert face-to-face meetings to virtual platforms. Participants to these virtual meetings were identified in an iterative process exploiting the professional networks of COMED partners. The main selection criteria were familiarity with the HTA ecosystem for MDs in late technology adopter countries with efforts for maintaining balanced geographical distribution of participants.

In April 2020 the list of issues and draught recommendations were presented to a small group of HTA experts with familiarity on national pricing and reimbursement processes of MDs in CEE countries in a virtual focus group meeting. After a thorough discussion, the descriptions of issues and recommendations were improved based on the consensus of participants.

The recommendations were validated with a wider group of experts, including HTA and reimbursement decision makers from CEE countries in May and June 2020. The validation process started with a webinar to present the issues and recommendations to a wider group of experts. Then, participants of the focus group meeting and the webinar in addition to COMED consortium members were asked to provide written feedback on draught recommendations. Finally, a virtual interactive meeting was organised to discuss the written feedback and facilitate consensus among participants.

Overall, 31 experts outside from the COMED consortium representing 14 CEE countries (Bulgaria, Croatia, Czech Republic, Greece, Hungary, Malta, Poland, Romania, Russia, Serbia, Slovakia, Slovenia, Turkey and Ukraine) contributed to the deliberative process.

## Results

### Targeted Literature Review

A total of 563 records were identified by the literature search. After removal of duplicates and title-abstract screening, 20 articles were found to be eligible for this review. Integrating with the 8 extra articles identified by the snowball method, in total, 28 articles qualified for a full-text review, and 19 were considered eligible for the qualitative synthesis. The numbers of identified, screened and excluded papers are shown in the flowchart in Supplementary Material 2.

In the targeted literature review process, 33 issues were identified with potential relevance for medical device HTA in late adopter European countries. The 33 issues were merged and reduced to 9 important issues with special relevance to CEE countries after iterative brainstorming sessions (Supplementary Material 3). The 9 issues were grouped to clinical value assessment, economic value assessment and HTA process.

### Challenges and Recommendations for HTA of MDs in Late Adopter Countries

#### Issue 1: Clinical Value Assessment

##### Challenge 1.1: Lower level of evidence for MDs

Evidence base is relatively limited for the majority of MDs compared with pharmaceuticals due to several reasons. Regulatory agencies do not mandate confirmatory efficacy and safety data on MDs from randomised clinical trials (RCTs) (1, 9–14). Hence, a substantial proportion of HTA reports rely on surrogate outcomes in estimating the health gain, often without proper validation (15). Although the recent EU regulation (2017/745 of the European Parliament) (16) made RCTs mandatory for Class IIb and III MDs, it is unforeseen whether the evidence base of MDs can be increased to the level of pharmaceutical, partly because blinding and proper randomisation cannot be implemented for all MDs (2). In several cases clinical trials for MDs are not adequately powered (i.e., sample size is too small) and/or the follow-up period is too short for performing HTA analysis (15, 17–19). Finally, MDs are often introduced into clinical practise quickly, especially in high income countries with great market potential, often even before the initiation of clinical trials (17). Then, when the MD is introduced in late adopter CEE countries, it is no longer feasible, or ethically justifiable to carry out RCTs (17).

*Recommendation #1.1.a: Use relative effectiveness and safety assessment from joint EU work or use rigorous relative assessment from other jurisdictions*. Relative effectiveness and safety assessments (RESAs) from the joint work of EU member states should be the starting point for CEE countries to judge the clinical effectiveness and safety of a MD (20). If this is not available, rigorous RESAs from other early adopter countries may be transferred to CEE countries. When relying on results from other countries, the absolute risk reduction should be adjusted based on the local baseline risk and uncertainty of the parameters should be explored with sensitivity analysis. Iterative reassessment should be considered when better evidence becomes available.

*Recommendation #1.1.b: Rely on real-world evidence, when evidence from explanatory randomised clinical trials is limited*. If evidence from explanatory RCTs is limited, the best available evidence should be synthetized with special attention to real-world evidence (RWE) (21). Comparative observational studies, patient registries or claims databases are examples of real-world data (RWD) that can produce RWE on the effectiveness and safety of MDs. However, appropriate methods of bias-adjustment (e.g. counterfactual effect estimation) need to be applied (22). RWE from early adopter countries may be transferred to CEE countries.

*Recommendation #1.1.c: Consider coverage with evidence development, when the scientific evidence from randomised clinical trials and real-world is premature*. A possible solution for the decision-makers to deal with uncertainty is to apply coverage with evidence development (CED) schemes in a specified patient population tracked over a defined period of time under explicit requirement of further evidence generation, where the level or continuation of reimbursement is based on the clinical and economic outcomes achieved. Due to the administrative and financial burden of implementing CED schemes in countries with limited resources, they should be applied for MDs in areas with high unmet medical need and public health priority. CED schemes from early adopter countries may be transferred to CEE countries.

##### Challenge 1.2: Limited transferability of real-world data and real-world evidence from foreign countries

Even though RCTs may not reflect the effectiveness of MDs in the real-world, they still represent the gold standard of evidence generation for new technologies (23). However, more and more stakeholders are exploring new areas of evidence generation based on real-world data since rigour for its collection has improved and standardisation has been initiated (24). While RWD can increase the evidence base of health technologies, RWD cannot completely replace RCTs. For quality assessment purposes RWD and the derived RWE are virtually inseparable (25–27). Due to limited availability of efficacy data from explanatory RCTs and the methodological principles intrinsic to RCTs (28, 29), the relative weight of RWD in the evidence generation of MDs is greater compared with pharmaceuticals.

In CEE countries where MDs are introduced in later life cycle stages, HTA of MDs could be accelerated through adapting RWD to local jurisdictions. However, CEE countries have limited power to access the original RWD from other countries (10, 30). Therefore, transferability assessment cannot focus on RWD, but on RWE.

It is highly important to note, that RWE is more subject to local factors compared with protocol driven RCTs, partly because the benefit of MDs is dependent on capacity constraints for example in the follow-up care and heterogeneity of patient pathways. Consequently, transferability of RWE is more limited than transferability of evidence from RCTs.

*Recommendation #1.2: Explore the feasibility of transferring RWE to late technology adopter countries in a stepwise approach*. In order to overcome the uncertain quality and local relevance of RWD, a stepwise approach is recommended to explore the feasibility of transferring international RWE to CEE countries. The first step should be the systematic search and collection of potentially relevant RWEs, followed by an evaluation of the equivalence of technologies presented in the study of origin and local context. As a result, studies in which the technologies have no major differences should be channelled into further assessment. In the next step, the quality of evidence should be evaluated by using quality assessment tools, such as GRADE (31, 32), ROBINS-I (33) or the ISPOR initiative on RWE transparency (34) and include only studies with appropriate quality for quantitative synthesis (e.g., meta-analysis). As a last step to guarantee applicability of evidence in local jurisdiction, use of a transferability checklist [e.g., EUnetHTA Adaptation Toolkit (35)] should be considered to evaluate the variation in patient population, medical practise and health systems. In CEE countries with capacity constraints in the follow-up care and heterogeneous patient pathways, highly conservative effectiveness estimates should be applied. On the other hand, limited variability in the real-world outcomes across centres in the country of origin indicates less dependence on local factors, and consequently increased transferability of high-quality RWE with appropriate comparator can be assumed to late technology adopter countries.

##### Challenge 1.3: Limited transferability of surrogate endpoints

Surrogate endpoints (36) are intended to replace patient/clinical relevant final endpoints, providing the possibility of using indirect measurement in cases when direct measurement of long-term clinical effects is not feasible. However, reliability of surrogate outcomes in predicting effects on clinically meaningful outcomes has to be validated, i.e., the treatment effect on a surrogate endpoint needs to be predictive of the treatment effect on the final clinical outcomes (15, 37). Due to differences in populations and health care systems, surrogate outcome predictions from other jurisdictions may not be directly transferable to CEE countries without adjustment.

*Recommendation #1.3: Reuse internationally validated surrogate endpoints with extensive sensitivity analyses*. Reuse of internationally validated surrogate endpoints with extensive sensitivity analyses is highly recommended in countries with limited HTA resources. The use of surrogates should be limited to HTAs using the same intervention, class of technology and comparator as the validation. If modelled to determine cost-effectiveness, the uncertainty of the extrapolation of surrogates to final outcomes should be fully considered.

If the surrogate endpoint is endorsed by major regulatory agencies (e.g., FDA) or HTA bodies (e.g., NICE, HAS, IQWIG, CADTH) it should be accepted in the base case scenario. International predictions for long-term outcomes should be complemented with extensive sensitivity analysis by considering a wide range of scenarios based on local factors.

If major regulatory agencies or HTA bodies have not endorsed the surrogate outcome, consider the best available scientific evidence with special focus on surrogates validated by international clinical societies for local assessment with more conservative estimation of long-term benefits.

##### Challenge 1.4: Learning curve

The effectiveness of many MDs depends not only on the device itself, but also on how it is used (17, 18, 38). A learning curve is often associated with a MD, as user skills and training with the new technology can have important implications on clinical outcomes (1, 2, 13, 29, 39, 40). Central and Eastern European countries are generally late adopters, so evidence on learning curves from more developed health systems may be available and can be adapted for the assessment of a MD in a local context.

On the other hand, health care provision in CEE countries is traditionally less standardised (i.e., protocol driven) and due to resource constraints, “*ad hoc*” solutions are more considered. During the launch period of new MDs, the support of manufacturers in providing training or collecting outcomes data in patient registries is often less intensive in countries with limited market potential. These factors are expected to contribute to longer learning curves, which can result in inferior outcome initially due to incremental safety problems or treatment failures in CEE compared with more advanced health care systems.

*Recommendation #1.4: In the introductory period of MDs consider inferior effectiveness and safety (1) based on learning curves from other countries (2) and by using Bayesian approach*. Learning curve should be considered in the HTA of MDs. If local evidence is not available, the transferability of learning curve results from early adopter countries should be assessed. In more resource constrained health systems in CEE countries, differences in the length and shape of learning curves need to be considered, and sensitivity analysis should be used to assess different plausible scenarios. Another recommended method to adjust the expected learning curves is the use of expert opinion on the magnitude of inferior outcomes due to incremental safety problems or complication rates in CEE countries, channelled in the evaluation process using a Bayesian approach with informative priors. The estimations can be updated once local evidence is available. Shorter learning curves can be assumed, if tailor-made manufacturer support is available for the medical institution, for health care professionals, or for patients (e.g., in case of self-administered MDs, such as wearables) during the introductory period.

##### Challenge 1.5: Centre effect

Several external factors related to the ability of health care professionals and institutes can influence the clinical effectiveness of MDs. National health care systems are organised into different centres, which obviously differ from each other. The centre effect should be considered in a hierarchical order, as substantial variation in provider skills may reside at the individual health care professional, team or hospital level (13, 18, 41).

The institutional environment like the placement and size of the centre, has dual impact on outcomes delivered by the medical device. On one hand, the limited number of patients in smaller centres can limit the ability of individuals to acquire appropriate competencies, which extends the learning curve preventing the maintenance of required skills to achieve maximum performance. On the other hand, large volume and diversity of services can influence the ability of institutes to exploit maximum effectiveness of MDs. In this regard, economies of scale in large specialised centres accelerates the adoption of MDs (42) and allows institutes to employ more specialised operators and ancillary staff, which ultimately improves outcomes and reduces the average cost per case. In larger centres, economies of scope with broader portfolio of services may improve health outcomes in complicated cases (e.g., multimorbid patients) by ensuring access to specialty services.

*Recommendation #1.5: Consider the relative effectiveness and safety of MDs in large volume centres with licenced health care professionals*. Since scale and scope of services matter, primary judgement on the relative effectiveness and safety of MDs should focus on large volume centres with licenced health care professionals for a specific device. By introducing a threshold on a minimum number of patients to be treated at the institution and individual operator level, limiting the number of centres and by defining quality standards, the outcomes variation related to centre effect can be reduced. These quality assurance tools may be transferred from early adopter countries. Furthermore, by initiating a medical licencing system, the maintenance of required expertise can be guaranteed. In countries with decentralised health care system and small volume centres, inferior effectiveness should be assumed, especially where licenced health care professionals for the specific medical device are not available. Additionally, an HTA model grounded outcome-based risk sharing system can directly incentivize health outcome improvement.

#### Issue 2: Economic Value Assessment

##### Challenge 2.1: Cost calculation of complex medical devices for cost-effectiveness and budget impact analyses

The price transparency of complex MDs is limited due to several factors. At first manufacturers of MDs can collect income through different channels, and the prices for the equipment, consumables and maintenance depend on strategic considerations by manufacturers. At health care institutes the equipment represents fixed costs and booked among capital costs, while consumables and *ad hoc* maintenance expenses are variable costs and booked among operating costs. As the average cost is dependent on the economies of scale, it is not easy to calculate the actual cost per intervention of complex MDs with high upfront costs and unpredictable maintenance costs (43).

In addition, differences in local production functions among hospitals further increases the variability of actual cost, especially since resource utilisation within a hospital can also change over time due to organisational changes and experience of health care professionals. Finally, cost of necessary initial and continuous training of health care professionals should also be taken into account, when calculating the cost per case of complex MDs. On the other hand, the availability of public health care budgets to cover new technologies—including MDs—is more limited in CEE countries, and due to less advanced control mechanism, prices of MDs in CEE countries may even be higher than in more affluent WE countries.

*Recommendation #2.1: HTA for MDs should be considered primarily for national reimbursement decisions or centralised procurement by taking into account average expected payments, rather than actual costs*. In countries with limited HTA resources, HTA for MDs should primarily focus on population level policies such as national reimbursement or centralised procurement decisions. To support the evidence base of national decisions, cost calculations should not depend on non-transparent price components, provider specific production functions and economies of scale. Consequently, instead of actual costs, the payment per case requested by health care providers or paid by health care payers (e.g., fees or charges) should be used in economic evaluations and budget impact analyses. If the focus of the national HTA assessment is the procedure completed by the MD rather than the MD itself, the payment for the procedure should be used, which covers costs of the MD, institutional cost and personnel cost as well.

Due to scarcity of HTA capacities at the local level, MD procurement decisions by local institutions may not be supported by full scope HTA reports. Still, decision-makers at local hospitals should be able to judge whether the payment per case by health care payers covers their expected local cost per case. This necessitates return on investment and financial sustainability calculation, which should be based on actual cost calculations by taking into account local production functions and economies of scale. On the other hand, if a CEE country still prefers hospital based HTA in addition to national assessments, each hospital should be able to calculate the actual cost of MDs per case from their own perspective, which may not be transferable to other hospitals.

##### Challenge 2.2: Limited transferability of economic evaluation of MDs

Being late adopters of MDs, CEE countries can potentially rely on HTA recommendations of early adopter countries. However, even if a given technology considered cost-effective in a WE country, it does not justify a positive recommendation in more resource constrained CEE countries, partly due to differences in the cost-effectiveness thresholds (44, 45). In addition, the variability in the incremental cost-effectiveness ratio of health technologies within different jurisdictions is documented in several publications (46–48). Therefore, transferring results and conclusions of an economic evaluation conducted in a higher income country along with the differences in patient population, comparator, patient pathways, outcomes, resource utilisation and unit costs and potential differences in the device itself represents several challenges (11, 49).

*Recommendation #2.2: Adapt international economic models from early technology adopter countries after transferability assessment*. In order to overcome the challenges of economic model adaptation from early adopter to CEE countries, a stepwise process is recommended. As a first step, a transferability of the model should be evaluated in particular to local relevance of the model concept and focusing on comparator, patient pathways and long-term outcome estimation. After adjusting the model to local circumstances, the use of country specific costs as a mandatory step is recommended. This should incorporate local patient pathways, average resource use in large centres and local unit cost. During the estimation of health outcome, as much local data as possible should be included to increase the relevance of the prediction. Discount rate, time-horizon of the assessment and other necessary input parameters should be adjusted according to the local methodological guideline. As a last step, a comprehensive sensitivity analysis is recommended to explore the effect of parameter uncertainty.

#### Issue 3: HTA Process

##### Challenge 3.1: Frequent product modifications and dynamic pricing

Unlike drugs, MDs often undergo several product modifications over time, which may have an impact on health gain, costs and patient experience (50). In late technology adopter CEE countries, often modified versions of MDs are introduced and have to be evaluated by HTA. In addition, prices of MDs more often change over time due to the market entry of new products (17, 39), iterative incremental developments over time (1, 2, 18, 39), and flexible procurement practises (17, 39). This raises the question of whether and when these small improvements have to be re-evaluated.

*Recommendation #3.1: HTA should not be performed for a particular version of a MD, but for the group of devices with the same (or similar) characteristics*. Full HTA is needed to support the initial national reimbursement decision or centralised procurement of a new MD. However, no HTA (or only fast track HTA) is necessary if (1) a different manufacturer introduces a similar MD with non-inferiority and no price increase; (2) the price of a reimbursed MD is reduced; (3) an improved MD emerges without price increase. A full HTA is still recommended, if total cost of the procedures with a modified MD is increased due to added value. A conservative approach should be followed when assessing the added clinical value of modified MDs.

##### Challenge 3.2: Diverse and numerous clinical indications of MDs

Several complex medical devices such as radiotherapy, robotic surgery, imaging diagnostics, can be used for heterogeneous patient populations in multiple indications. However, in countries with limited HTA resources it is not possible to conduct full scope HTA in all different indications.

*Recommendation #3.2: Full scope HTA may not be necessary in each potential indication, cost-effectiveness results in the most prevalent indications can be generalised to indications with similar expected health benefits*. After exploring all potential indications, the list should be narrowed to those indications which are candidates for reimbursement by excluding indications with no assumed incremental health gain or with low public priority. Full scope HTA with detailed cost-effectiveness analysis has to be conducted in the most prevalent indications. If the MD is cost-effective in the most prevalent indications, in case of limited HTA capacities, it is an acceptable compromise to generalise cost-effectiveness evidence in the most prevalent indications to other indications with similar estimated health gain. However, budget impact analysis is needed for the entire target patient population eligible for public coverage.

The recommendations described above are summarised in Table 1.

**Table 1:** Consensus recommendations related to selected challenges of medical device HTA in late adopter countries.

**Major issues**	**Summary of challenges and recommendations**
1. Clinical value assessment (effectiveness and safety)	1.1 Lower level of evidence for MDs - Use relative effectiveness and safety assessment from joint EU work or use rigorous relative assessment from other jurisdictions - Rely on real-world evidence, when evidence from explanatory randomised clinical trials is limited - Consider coverage with evidence development, when the scientific evidence from randomised clinical trials and real-world is premature
	1.2 Limited transferability of real-world data and real-world evidence from foreign countries - Explore the feasibility of transferring real-world evidence to late technology adopter countries in a stepwise approach
	1.3 Limited transferability of surrogate endpoints - Reuse internationally validated surrogate endpoints with extensive sensitivity analyses
	1.4 Learning curve - In the introductory period of medical devices consider inferior effectiveness and safety (1) based on learning curves from other countries (2) and by using Bayesian approach
	1.5 Centre effect - Consider the relative effectiveness and safety of medical devices in large volume centres with licenced health care professionals
2. Economic value assessment (cost calculation, cost-effectiveness, budget impact)	2.1 Cost calculation of complex medical devices for cost-effectiveness and budget impact analyses - HTA for medical devices should be considered primarily for national reimbursement decisions or centralised procurement by taking into account average expected payments (e.g., fee or charges) rather than actual costs
	2.2 Limited transferability of economic evaluation of medical devices - Adapt international economic models from early technology adopter countries after transferability assessment
3. HTA process	3.1 Frequent product modifications and dynamic pricing - HTA should not be performed for a particular version of a medical device, but for the group of devices with the same (or similar) characteristics
	3.2 Diverse and numerous clinical indications of medical devices - Full scope HTA may not be necessary in each potential indication, cost-effectiveness results in the most prevalent indications can be generalised to indications with similar expected health benefits

*MDs, medical devices; HTA, health technology assessment*.

## Discussion

Those European countries which are late adopters of new technologies usually have more limited resources not only for covering high-cost innovative technologies from public resources, but also for conducting HTA to substantiate their health policy decisions with appropriate evidence base. However, delays in the uptake of new technologies create an opportunity to benefit from HTA methods and reports generated in early technology adopter countries. Therefore, considering the appropriate reuse of international HTA materials, late technology adopter countries can still implement HTA, even for MDs, which have more limited evidence base compared with pharmaceuticals.

As a general recommendation, CEE countries should be encouraged to contribute to joint work to extend the evidence base of MDs, including generation of RWE, validation of surrogate endpoints and exploratory research on learning curves. The increased participation of CEE centres in international collaborative research projects can improve the knowledge on the transferability of scientific evidence related to MDs. CED schemes should also be considered in CEE, however, further research is needed on which elements of CED schemes from early adopter countries are transferable to late adopter countries.

Recommendations given in this report may provide guidance for policy-makers on how standards for HTA of MDs should be improved in lower income countries. Although adaptation of these recommendations should remain in national competencies, if these proposals are translated into routine practise, manufacturers will have more clarity on what they need to deliver to facilitate the market access of their new MDs in countries with less market potential. Recommendations are not equally relevant for all different types of MDs.

Ultimately, we hope that applying these recommendations can lead to better care for patients with greater need for health improvement in more deprived European countries by reducing the opportunity cost of inappropriate coverage decisions.

Although in our guidance we focused mainly on lower income European countries especially in CEE, these recommendations can be valid to any lower income country outside Europe, which are also late adopters of MDs due to their limited market potential, and also to any small country, which have limited human and financial resources to support coverage decisions with HTA evidence.

It should be noted that our recommendations are based primarily on the opinion of relatively small-sized expert group, which is the most important limitation of our study. However, focusing on commonalities and not differences across countries with highly experienced HTA professionals in the deliberative process facilitated the replicability of our conclusions. Still, our recommendations cannot be equally generalizable to all different categories of MDs. Hence future research, especially at the national level targeting different types of MDs, should test the appropriateness of our recommendations, which may necessitate additional recommendations for specific groups of MDs.

Overall, our guidance should be viewed only as a first step in a multi-stakeholder dialogue about HTA practises of MDs in lower income European countries, which can be strengthened by voluntary regional collaboration in HTA.

## Data Availability Statement

The original contributions generated in the study are included in the article/Supplementary Material, further inquiries can be directed to the corresponding author/s.

## Author Contributions

RD-B, SK, AZ, and ZK iteratively designed the study methodology, developed the initial guidance, reviewed and edited the materials. RD-B and SK completed on the targeted literature review. AZ and ZK undertook conceptualisation and supervised the entire project. MD, OC, RodT, CB, AT, and RosT developed those recommendations in the COMED consortium, which had to be addressed from the perspective of CEE countries interactively with the Syreon team. MD, OC, RodT, CB, and MA extensively commented each version of the manuscript. MD contributed to the conceptual aspects from the initial phase, took major role in the virtual workshops, contributed to the parts about coverage with evidence development. OC and RodT contributed to the parts about surrogate endpoints. CB contributed the parts related to early assessment of medical devices. MA contributed to the parts about learning curve. AT and RosT are the thought leaders of COMED consortium, supported the transferability exercise throughout the project with presentations and extensive review of methodological aspects and draught reports. MN, MH, OM, JY, GP, MH-V, OP, and LL took part in the validation process, reviewed the recommendations from their own stakeholder and geographical perspectives, and commented extensively each manuscript version. All authors approved the submitted paper.

## Conflict of Interest

RD-B, SK, AZ, and ZK were employed by Syreon Research Institute. MH is self-employed in HTA/EBM Consulting Centre and has not received any remuneration of funding from the COMED project, nor has any commercial or financial relationship that could be construed as a potential conflict of interest. The remaining authors declare that the research was conducted in the absence of any commercial or financial relationships that could be construed as a potential conflict of interest.
