# Supplementary material for: Quo Vadis HTA for Medical Devices in Central and Eastern Europe? Recommendations to Address Methodological Challenges
Source: Front Public Health. 2021 Jan 8;8:612410. doi: 10.3389/fpubh.2020.612410 (PMC7820783; doi:10.3389/fpubh.2020.612410)
Supplement: Supplementary file 1 [file Table_1.docx]

**Supplementary material**

**Supplementary material 1** Search strategy of the targeted literature review

| **SCOPUS** | Specification | Search terms | Date | Number of hits |
| --- | --- | --- | --- | --- |
| #1 |  | ( TITLE-ABS-KEY ( assessment ) OR TITLE-ABS-KEY ( evaluation ) OR TITLE-ABS-KEY ( appraisal )OR TITLE-ABS-KEY ( estimation ) ) | 28/01/2020 | 7,567,627 |
| #2 |  | ( TITLE-ABS-KEY ( barriers ) OR TITLE-ABS-KEY ( challenge ) ) | 28/01/2020 | 2,328,929 |
| #3 |  | TITLE-ABS-KEY ( "medical device" ) | 28/01/2020 | 56,408 |
| #4 | Combined search | #1 AND #2 AND #3 | 28/01/2020 | 963 |
| #5 | Limited to | #4 AND Filters: English and Medical Journal | 28/01/2020 | **563** |

**Supplementary material 2** Flow diagram of targeted literature review for challenges related to MD assessment

Records identified through Scopus
(n=563)

Records screened
(n = 563)

Records excluded

Not relevant for the research question (n = 543)

Full-text articles assessed for eligibility (n= 28)

Records excluded
(n =9)

- Full text not available

(n = 1)

- Challenges not related to MD assessment (n = 8)

Studies included in the qualitative synthesis
**(n = 19)**

Extra articles identified with snowball method
(n= 8)

**Identification**

**Screening**

**Eligibility**

**Inclusion**

**usion**

**Supplementary material 3** The 33 issues found in the literature were merged and reduced to 9 important issues with special relevance to late technology adopter countries

| **No** | **Issues found in the literature** | **Judgement** | **Final issue category** |
| --- | --- | --- | --- |
| 1 | RCT design not feasible | overlap, merged | 1.1 Lower level of evidence for MDs |
| 2 | Shorter-term studies | overlap, merged | 1.1 Lower level of evidence for MDs |
| 3 | Small sample sizes in RCTs | overlap, merged | 1.1 Lower level of evidence for MDs |
| 4 | Scarce evidence due regulatory requirements | overlap, merged | 1.1 Lower level of evidence for MDs |
| 5 | Frequent product modifications | overlap, merged | 3.1 Frequent product modifications and dynamic pricing |
| 6 | Rapid diffusion of MDs even before sufficient evidence is available | overlap, merged | 1.1 Lower level of evidence for MDs |
| 7 | Procedures of market access of MDs do not encourage data generation | overlap, merged | 1.1 Lower level of evidence for MDs |
| 8 | Lack of procedures to incentivize further research | overlap, merged | 1.1 Lower level of evidence for MDs |
| 9 | Learning-curve effect | overlap, merged | 1.4 Learning curve |
| 10 | Role of manufacturer support in effectiveness | overlap, merged | 1.4 Learning curve |
| 11 | Incremental device innovation (similar to frequent product modification) | overlap, merged | 3.1 Frequent product modifications and dynamic pricing |
| 12 | Centre effect - causing limited generalizability of the evidence | overlap, merged | 1.5 Centre effect |
| 13 | Dynamic pricing might affect cost-effectiveness | overlap, merged | 3.1 Frequent product modifications and dynamic pricing |
| 14 | Limitations in generation of high-level evidence | overlap, merged | 1.1 Lower level of evidence for MDs |
| 15 | Lack of specific guidance for different types of MDs | excluded because non-specific to our scope | - |
| 16 | Lack of guidance for complex technologies | unique category | 3.2 Diverse and numerous clinical indications of MDs |
| 17 | Lack of RWD quality assessment tool | overlap, merged | 1.2 Limited transferability of RWD and RWE from foreign countries |
| 18 | Expertise gap in HTA appraisal | excluded because non-specific to our scope | - |
| 19 | Insufficient resources in HTA appraisal | excluded because non-specific to our scope | - |
| 20 | Upfront irrecoverable costs | overlap, merged | 2.1 Cost calculation of complex MDs for cost-effectiveness and budget impact analysis |
| 21 | Separation of decision-making bodies from research funding bodies | excluded because non-specific to our scope | - |
| 22 | Missing regulation of the market access of 'fast-followers' | excluded because non-specific to our scope | - |
| 23 | Heterogeneity in MD evaluation across Europe | excluded because non-specific to our scope | - |
| 24 | Incoherent device classification across countries | excluded because non-specific to our scope | - |
| 25 | Heterogeneity of device licensing | excluded because non-specific to our scope | - |
| 26 | Effectiveness can be influenced by hospitals capability to reorganize their services | overlap, merged | 1.5 Centre effect |
| 27 | Device-operator interaction: variation in provider skills | overlap, merged | 1.5 Centre effect |
| 28 | Non-generalizable MD evidence - intervention | overlap, merged | 1.2 Limited transferability of RWD and RWE from foreign countries;  1.3 Limited transferability of surrogate endpoints |
| 29 | Non-generalizable MD evidence - population | overlap, merged | 2.2 Limited transferability of economic evaluation of MDs |
| 30 | Non-generalizable MD evidence - comparator | overlap, merged | 2.2 Limited transferability of economic evaluation of MDs |
| 31 | Non-generalizable MD evidence - cost | overlap, merged | 2.1 Cost calculation of complex MDs for cost-effectiveness and budget impact analysis |
| 32 | Inadequacies in reporting evidence | overlap, merged | 1.2 Limited transferability of RWD and RWE from foreign countries |
| 33 | No local data for a model adaptation | excluded because non-specific to our scope | - |

*MD - medical device; RWD – real world data; RWE – real world evidence*
